# Morphological Characterization of Turkish Ambling Horses

**DOI:** 10.3390/ani16182813

**Published:** 2026-09-08

**Authors:** Burcu Bayramoğlu, Yahya Tuncay Tuna

**Affiliations:** Department of Animal Science, Faculty of Agriculture, Tekirdağ Namık Kemal University, Tekirdağ 59030, Türkiye; burcubayramoglu2013@gmail.com

**Keywords:** Turkish ambling horses, morphometric characterization, indigenous equine genetic resources, breeding origin, phenotypic variation

## Abstract

Morphometric information on ambling horses raised in Türkiye remains limited. This study aimed to characterize the morphometric traits of ambling horses according to breeding origin, age, and sex and to provide baseline phenotypic information on the studied population. A total of 102 naturally ambling horses were evaluated, including indigenous horses raised in Bursa (*n* = 28), indigenous horses raised in Nazilli, Aydın Province (*n* = 65), and Afghan-origin horses raised in Bursa (*n* = 9). Morphometric measurements of the head, body, and limbs were recorded, and the effects of breeding origin, age, and sex were analyzed using a general linear model. Significant differences among breeding origins were observed for several morphometric traits (*p* < 0.05). Afghan-origin horses generally exhibited higher values for several body measurements, particularly withers height, body length, chest depth, chest width, and heart girth, reflecting morphometric differences within the studied sample. Age- and sex-related differences were also observed for several morphometric traits. The strongest correlation was observed between CH and BH (*r* = 0.920, *p* < 0.001), followed by WH and BH (*r* = 0.873, *p* < 0.001) and WH and CH (*r* = 0.860, *p* < 0.001). ITH was also positively correlated with BH (*r* = 0.825), CH (*r* = 0.808), and WH (*r* = 0.744) (all *p* < 0.001). Overall, this study provides baseline morphometric information on ambling horses from different breeding origins raised in Türkiye and contributes to the phenotypic characterization of the studied population. However, because the Afghan-origin group included only nine horses and the distribution of animals among some groups was unbalanced, the observed differences should be interpreted with caution. Further studies involving larger and more balanced populations are needed to confirm these findings.

## Simple Summary

Ambling horses raised in Türkiye are recognized for their characteristic gait, but information on their body conformation remains limited. This study evaluated the morphometric characteristics of indigenous Turkish ambling horses raised in Bursa and Nazilli and Afghan-origin ambling horses raised in Bursa, with particular attention to differences associated with breeding origin, age, and sex. The ambling gait was an inclusion characteristic of the studied horses and was not quantitatively evaluated. Afghan-origin horses generally had larger body measurements than the indigenous Turkish populations, while several morphometric traits also differed according to age and sex. These findings provide baseline information for the phenotypic characterization of the studied ambling horse populations. However, because only nine Afghan-origin horses were included and the distribution of animals among some groups was unbalanced, the findings should be interpreted with caution. Studies involving larger and more balanced populations are needed to further characterize these horses.

## 1. Introduction

The horse has been one of the most important domesticated animal species throughout human history, playing a major role in transportation, agriculture, warfare, and human subsistence from approximately 5500 BC until the widespread mechanization of agriculture and transportation during the 1950s and 1960s [1,2,3].

Horse breeding has long been an important component of animal production in Türkiye [4]; however, the national horse population has declined markedly over the past century. According to FAO statistics, horse numbers decreased from approximately 309,000 in the early 2000s to 114,047 in 2017 and 70,360 in 2024 [5], largely in parallel with the mechanization of agriculture and transportation.

Recent advances in equine genetics have demonstrated that the ability to perform alternative gaits, including ambling gaits, is strongly associated with a mutation in the DMRT3 gene, which plays an important role in locomotor coordination and gait pattern [6,7]. Wutke et al. [8] identified the earliest known occurrence of this mutation in horse remains from England dated to approximately 850–900 AD, although this finding did not establish England as its definitive geographical origin. Genetic variation was not investigated in the present study; nevertheless, morphometric characterization of Turkish ambling horses may provide baseline phenotypic information for future studies integrating morphological, gait, and molecular genetic approaches.

In addition to the three basic gaits (walk, trot, and gallop), several alternative gaits, including the ambling gait, occur naturally in specific horse populations. Among Turkish horse populations, the Ayvacık Pony and the Canik Horse are recognized for their natural ambling gait [9,10,11]. Historical records indicate that gaited horses were introduced into Anatolia during the migration of Turkic peoples and were maintained throughout the Seljuk and Ottoman periods. Today, traditional ambling horse races continue under the Türkiye Traditional Sports Federation, contributing to the continuation of this cultural heritage [9].

Phenotypic characterization is an important step in the identification, conservation, and sustainable utilization of animal genetic resources [12]. Morphometric measurements are widely used to characterize horse populations and describe body conformation and may contribute to population classification and breed differentiation [13]. When age-related morphometric differences are interpreted, skeletal maturity should also be considered rather than chronological age alone [14]. A previous study on Turkish ambling horses reported mean values of 141.8 ± 0.62 cm for withers height, 140.1 ± 0.63 cm for croup height, 140.8 ± 0.86 cm for body length, 166.1 ± 0.95 cm for chest circumference, 64.3 ± 0.55 cm for chest depth, 17.9 ± 0.11 cm for cannon circumference, 57.3 ± 0.39 cm for head length, and 13.4 ± 0.12 cm for ear length [15]. Moreover, previous genetic research has indicated that Rahvan horses may form a genetically distinct population clustering separately from other native Turkish horse populations [16].

Despite their historical and cultural importance, Turkish ambling horses are threatened by declining population size, uncontrolled crossbreeding, and changes in breeding practices [17,18]. Nevertheless, conservation efforts by breeders, including the establishment of the Association for the Preservation and Development of Anatolian Horse Breeds in 1992 and the introduction of official ambling horse competition regulations in 1998, have contributed to maintaining these horse populations [17,18]. Seasonal ambling horse races also continue as an important component of traditional equestrian culture. The characterization and conservation of local horse populations remain important for preserving phenotypic and genetic diversity [17,18].

Despite increasing interest in the conservation and characterization of indigenous horse populations, comprehensive morphometric information on Turkish ambling horses remains limited. Therefore, the present study aimed to characterize the morphometric traits of indigenous Turkish ambling horses raised in Bursa and Nazilli and Afghan-origin ambling horses raised in Bursa. The effects of breeding origin, age, and sex on morphometric measurements were evaluated, and associations among the measured traits were examined using Pearson’s correlation analysis. The ambling gait was used as an inclusion characteristic of the study population but was not quantitatively evaluated. Accordingly, the present study was limited to morphometric characterization and did not assess gait performance, gait biomechanics, or genetic variation. The resulting data may provide baseline phenotypic information for future studies on the characterization and conservation of Turkish ambling horse populations.

## 2. Materials and Methods

The study was conducted in Bursa (40°11′50″ N, 29°03′44″ E) and the Nazilli district of Aydın Province (37°54′45″ N, 28°19′14″ E), Türkiye. A total of 102 naturally ambling horses were included in the study, with one horse sampled from each of 102 privately managed farms. Of these, 37 horses were sampled from farms in Bursa and 65 from farms in Nazilli. Among the horses sampled in Bursa, nine were of Afghan origin and had been imported from Afghanistan, whereas 28 were indigenous Turkish ambling horses. All 65 horses sampled in Nazilli were indigenous Turkish ambling horses.

**Figure 1:**
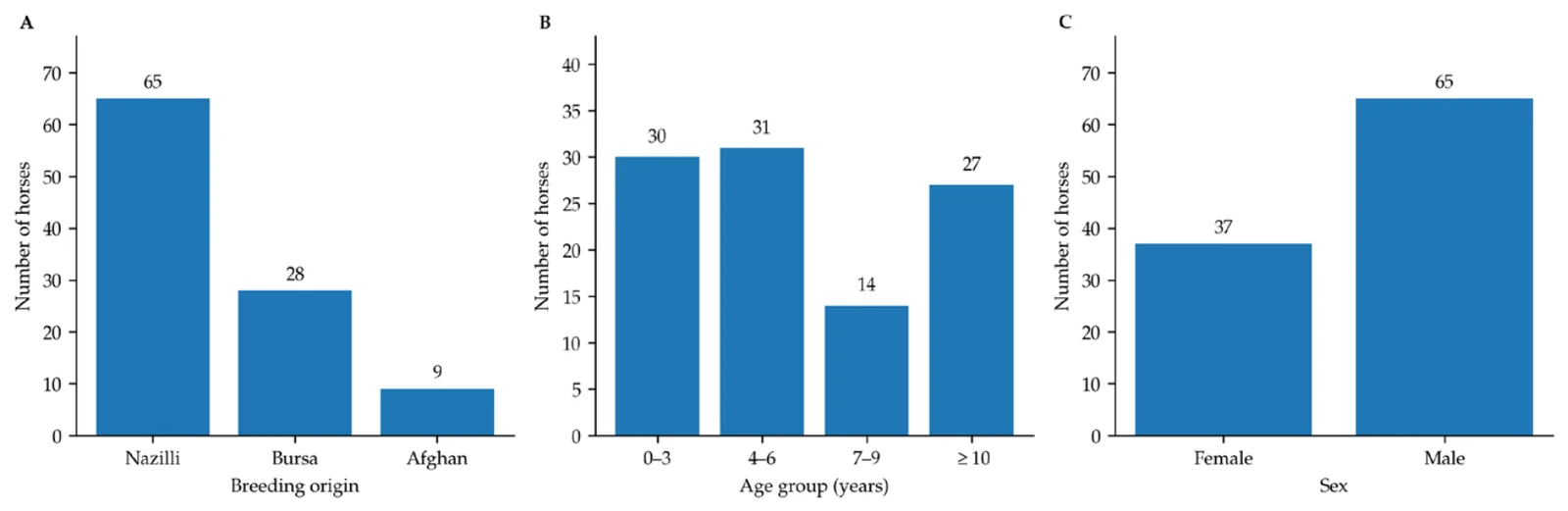
Distribution of Turkish ambling horses by breeding origin (**A**), age (**B**) and sex (**C**).

For the purposes of the present study, the term “origin” was used to distinguish the groups according to their breeding and geographical background rather than to indicate genetically defined populations or genotypes. Accordingly, the horses were classified into three breeding-origin groups for statistical evaluation: indigenous horses raised in Bursa (*n* = 28), indigenous horses raised in Nazilli (*n* = 65), and Afghan-origin horses raised in Bursa (*n* = 9). The study population consisted of 37 females and 65 males. Based on age, the horses were divided into four categories: 0–3 years (*n* = 30), 4–6 years (*n* = 31), 7–9 years (*n* = 14), and ≥10 years (*n* = 27) (Figure 1).

The present study was designed as a cross-sectional morphometric study; therefore, individual horses were measured only once, and their growth and development were not monitored longitudinally. The age of each horse at the time of morphometric measurement was determined based on information provided by the breeders.

The farms were visited on-site, and all morphometric measurements were obtained directly from the horses under field conditions using the same zoometric measurement procedures. The effects of breeding origin, age, and sex on the measured morphometric characteristics were subsequently evaluated.

Because of the small and uneven numbers of animals across some breeding origin × age × sex subgroups, interaction effects were not included in the statistical model to avoid potentially unreliable estimates (Table 1).

**Figure 2:**
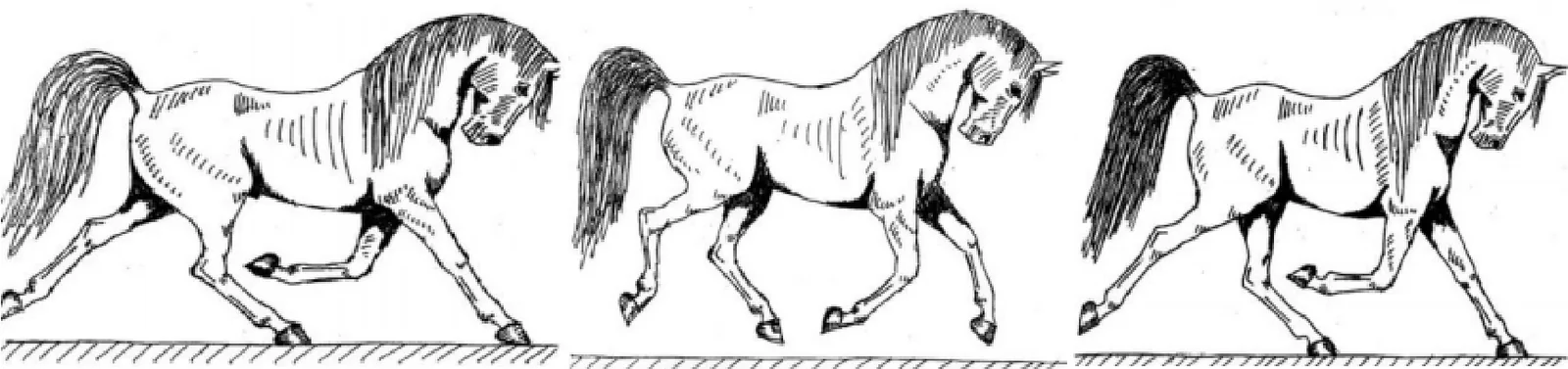
Demonstration of the ambling gait in horses [17].

The ambling gait was one of the characteristics used to identify and select the horses included in the study. All horses exhibited a natural ambling gait; however, gait characteristics were not quantitatively evaluated, and gait performance or biomechanics were not investigated. Therefore, the ambling gait should be considered an inclusion characteristic of the study population rather than a trait evaluated in the present study. The analyses and interpretations were limited to the morphometric characteristics of horses exhibiting this gait. The Turkish ambling horses included in this study naturally perform the ambling (rahvan) gait, an alternative gait found in only a few horse breeds. This gait is characterized by coordinated lateral limb movement, resulting in a smooth, comfortable ride with minimal vertical displacement. Consequently, it has traditionally been preferred for riding and long-distance travel in Türkiye (Figure 2) [19].

Accordingly, all horses included in the present study naturally performed the ambling (rahvan) gait, which constituted the principal criterion for their selection as the study material. According to Yücel [1], the terms rahvan, yorga, and eşkin describe closely related forms of ambling gait. While eşkin is of Turkish origin, rahvan is of Persian origin. The ambling gait is characterized as a two-beat gait in which the ipsilateral forelimb and hindlimb strike the ground simultaneously. As a result of this limb movement pattern, the horse’s body alternately sways to the right and left, producing a smooth and comfortable ride that minimizes rider fatigue. In this study, the term “origin” does not refer to the genetic background or genotype of the horses, but rather to their breeding or geographical origin (Bursa, Nazilli, or horses imported from Afghanistan). The common characteristic of all horses included in the study was their ambling gait, which constituted the primary criterion for their inclusion and evaluation.

Morphometric measurements were grouped into three categories: head, body, and limb traits. Head measurements included head length (HL) and head width (Hd W). Body measurements comprised withers height (WH), croup height (CH), back height (BH), tailhead height (TH), ischial tuberosity height (ITH), body length (BL), chest depth (CD), chest width (CW), heart girth (HG), ischial width (IW), and hip width (HW). Limb measurements included cannon girth (CG), cannon length (CL), hoof height (HH), and limb length (LL).

**Figure 3:**
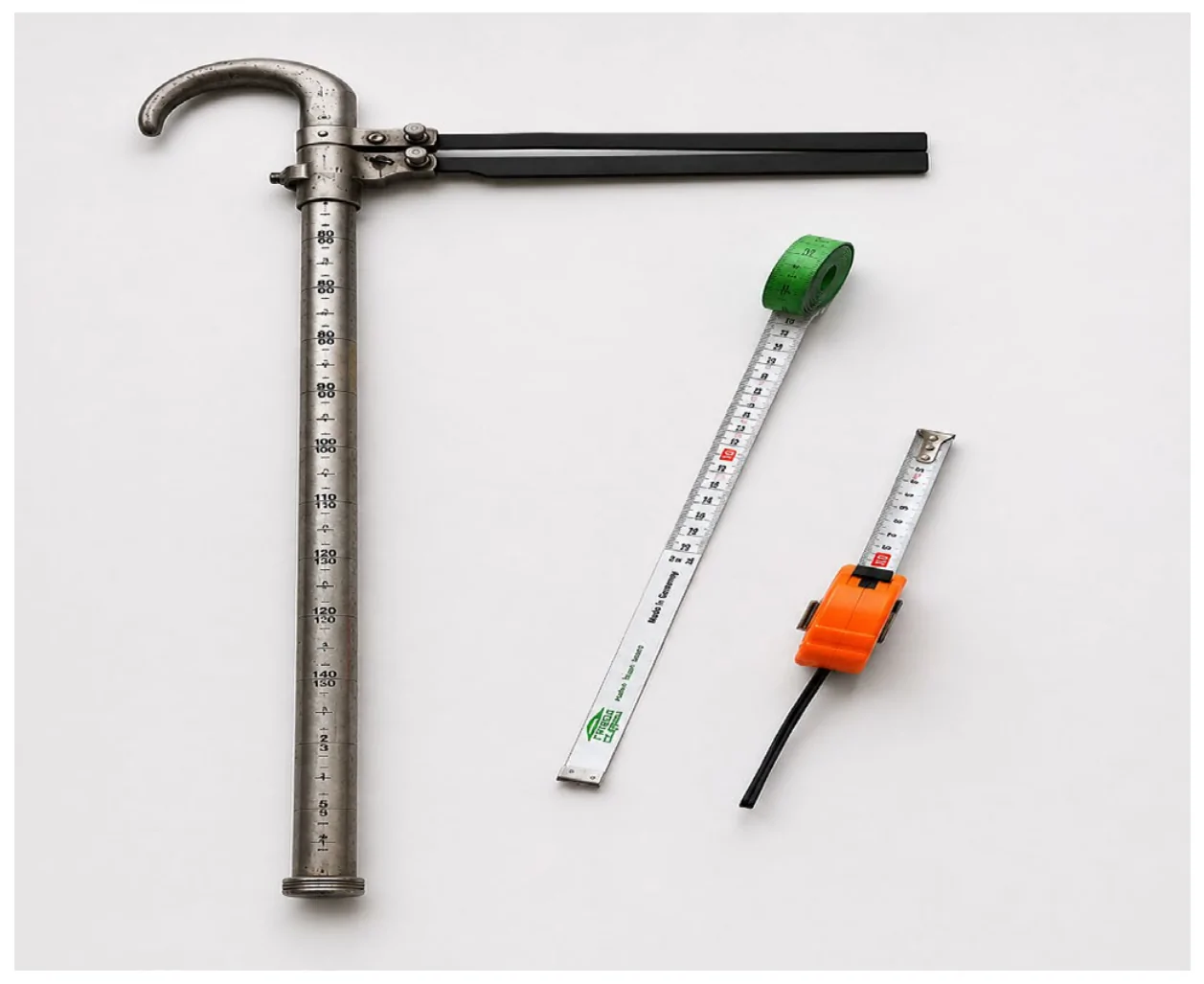
Instruments used for equine morphometric measurements.

Height measurements were obtained with a Lydtin (Hauptner, Solingen, Germany) measuring stick, whereas width, length, and circumference were recorded with a flexible measuring tape (Figure 3).

**Figure 4:**
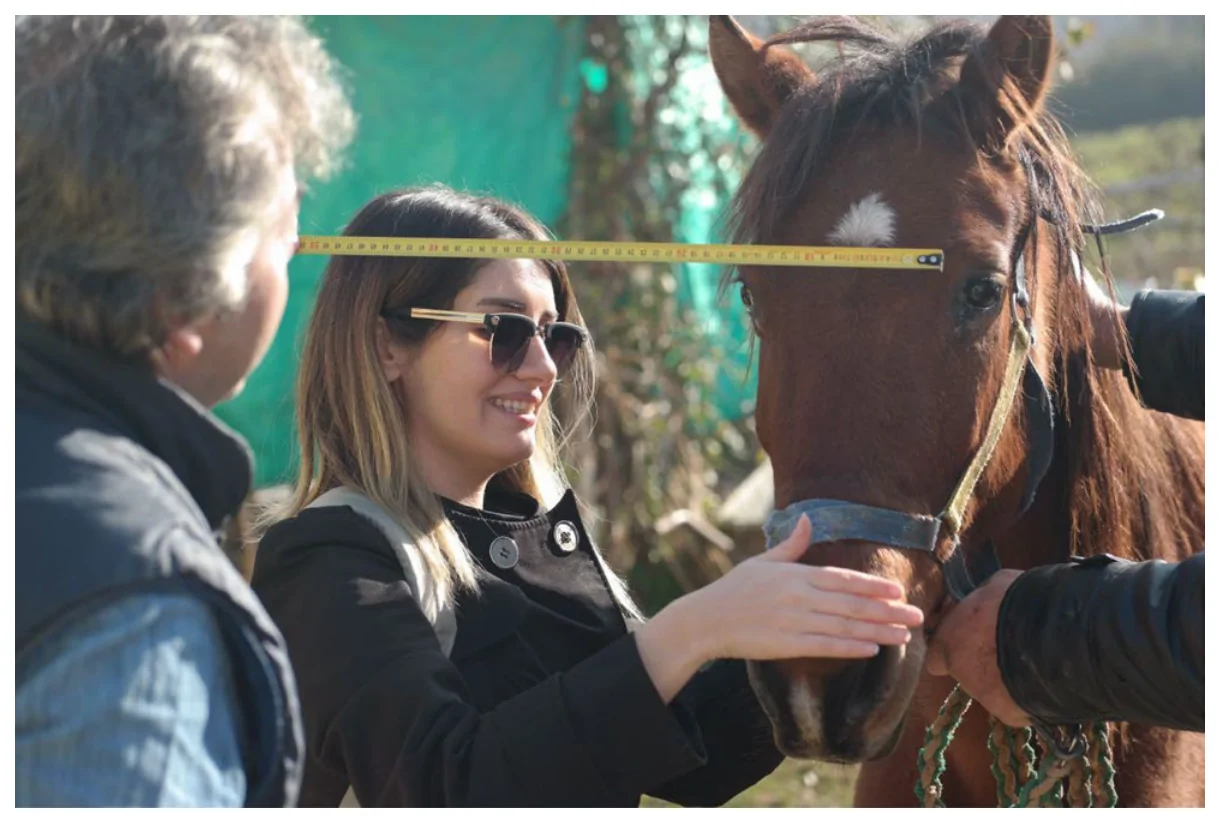
Researchers conducting morphometric measurements on a horse.

All morphometric measurements were performed by the same experienced researcher using a standardized measurement protocol to ensure consistency and reduce observer-related variation (Figure 4).

**Figure 5:**
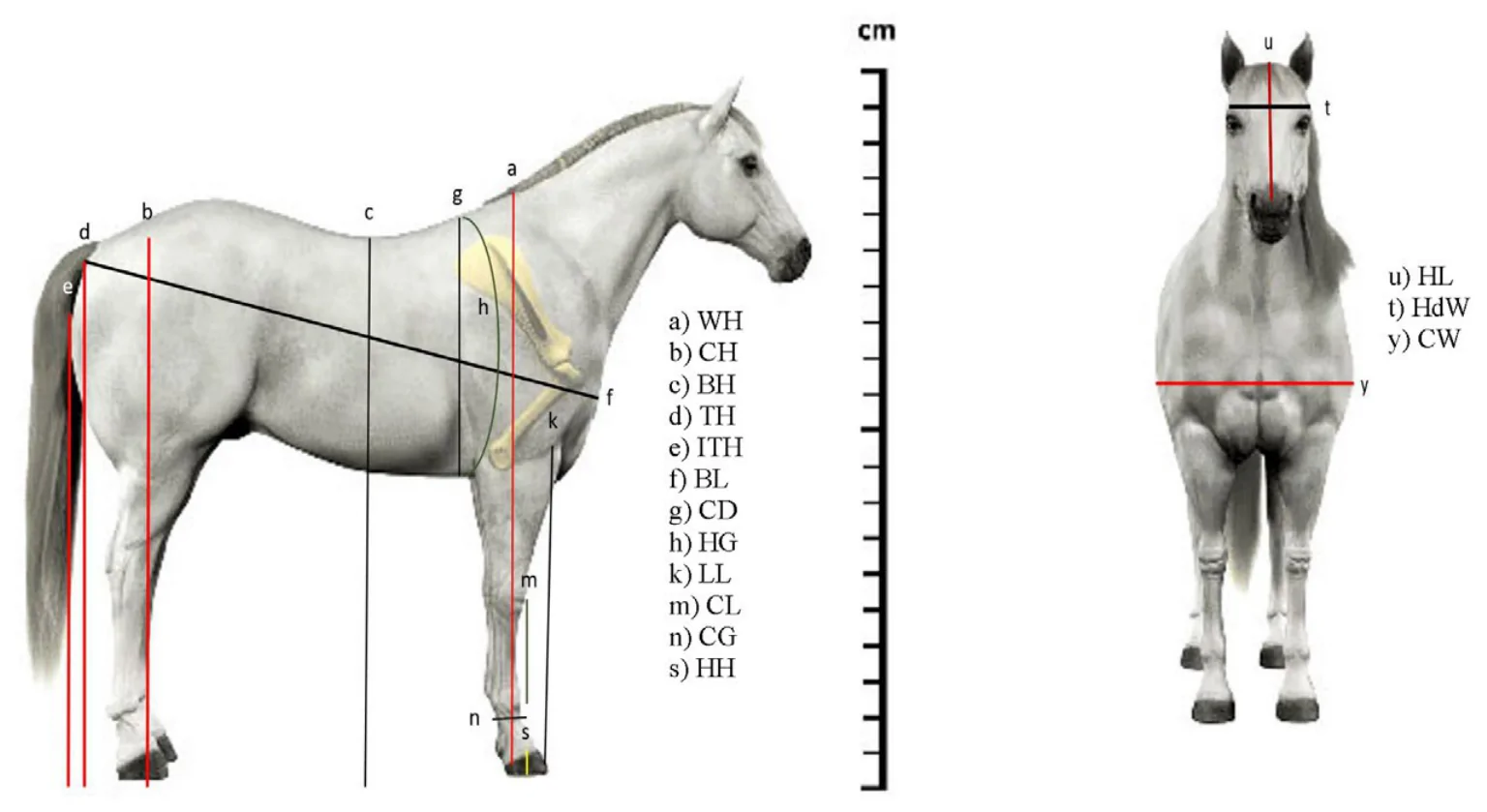
Schematic diagram of the morphometric measurements used in this study.

To ensure consistency between measurements, all morphometric measurements were performed within the same morning period using identical procedures (Figure 5). Horses from the Bursa and Nazilli populations were selected from herds managed under similar feeding, husbandry, management, and environmental conditions to minimize the influence of non-genetic factors on morphometric measurements. Each horse was measured only once. All morphometric measurements were obtained using standardized anatomical landmarks and measurement procedures to minimize measurement error and improve measurement consistency. Only clinically healthy horses with normal body condition and without any orthopedic abnormalities, conformational defects, or diseases that could influence morphometric measurements were included in the study. Pregnant and lactating mares were excluded. All morphometric measurements were taken from the left side of each horse. Measurements were obtained on a flat, level surface, with each horse standing squarely in a natural posture. As all horses were uniformly shod at the time of measurement, any potential effect of shoeing on height measurements was considered consistent across the study population. All horses were measured while shod with standard shoes, and height measurements included shoe thickness. Only horses with normal hoof conformation and routine hoof care were included in the study. Horses showing excessive hoof wear, hoof deformities, or abnormal hoof conditions that could affect hoof height measurements were excluded. All horses included in the study were actively used in ambling competitions and were therefore maintained under a regular shoeing schedule. Their horseshoes were routinely replaced at approximately 4–6-week intervals. Horses with unusually thick, corrective, excessively worn, or deformed shoes were not included in the study. Therefore, variation in shoeing was considered unlikely to represent a major source of bias in the morphometric measurements.

Phenotypic relationships among morphometric traits were assessed using Pearson’s correlation analysis in JMP^®^ Pro version 17 (SAS Institute Inc., Cary, NC, USA). Statistical significance was set at *p* < 0.05, *p* < 0.01, and *p* < 0.001.

Data were analyzed using a general linear model (GLM) in JMP, with breeding origin, age group, and sex included as model effects. Least-squares means (LS means) were estimated for each factor to obtain means adjusted for the other effects included in the model. Differences among LS means were evaluated using Tukey’s HSD multiple comparison test. Model assumptions were assessed prior to interpretation of the model results. Residual normality was evaluated using the Shapiro–Wilk test, and homogeneity of variance was assessed using Levene’s test. A *p*-value > 0.05 was considered indicative of acceptable residual normality and homogeneity of variance, respectively. Potential outliers were assessed using studentized residuals, with observations outside the range of −3 to +3 considered potential outliers.

$$\mathrm{Model}:\;Y_{ijkl}=\mu +K_{i}+Y_{j}+C_{k}+e_{ijkl}$$

where

$Y_{ijkl}$: observed value of the dependent variable;

μ: overall population mean;

$K_{i}$: fixed effect of the *i*th origin;

$Y_{j}$: fixed effect of the *i*th age;

$C_{k}$: fixed effect of sex;

$e_{ijkl}$: random residual error.

Head traits:

- HL (Head Length): Distance from the poll (occipital crest) to the most anterior point of the upper lip;
- HdW (Head Width): Maximum distance between the lateral borders of the zygomatic arches.

Body traits:

- WH (Withers Height): Vertical distance from the highest point of the withers to the ground;
- CH (Croup Height): Vertical distance from the highest point of the croup (*tuber sacrale*) to the ground;
- BH (Back Height): Vertical distance from the midpoint of the back to the ground;
- TH (Tailhead Height): Vertical distance from the tailhead (base of the tail) to the ground;
- ITH (Ischial Tuberosity Height): Vertical distance from the ischial tuberosity (pin bone) to the ground;
- BL (Body Length): Horizontal distance from the point of the shoulder (cranial aspect of the humerus) to the ischial tuberosity;
- CD (Chest Depth): Vertical distance from the highest point of the withers to the sternum immediately behind the forelimbs;
- CW (Chest Width): Horizontal distance between the medial borders of the shoulder joints immediately behind the forelimbs;
- HG (Heart Girth): Thoracic circumference measured immediately behind the withers and elbows;
- IW (Ischial Width): Distance between the left and right ischial tuberosities;
- HW (Hip Width): Distance between the left and right *tubera coxarum;*

Limb traits:

- CG (Cannon Girth): Circumference measured at the midpoint of the metacarpus (cannon bone) of the forelimb;
- CL (Cannon Length): Distance from the distal margin of the carpal joint to the proximal margin of the fetlock joint;
- HH (Hoof Height): Vertical distance from the coronary band to the ground at the dorsal hoof wall;
- LL (Leg Length): Vertical distance from the elbow to the ground.
- Morphometric measurements were obtained according to the standard equine morphometric procedures described by McManus et al. [20] and Pinto et al. [21].

## 3. Results

The mean withers height (WH) was 143.72 cm, while the mean body length (BL) and heart girth (HG) were 137.56 cm and 156.92 cm, respectively. Mean chest depth (CD) and chest width (CW) were 64.53 cm and 34.84 cm, respectively. Limb measurements averaged 18.81 cm for cannon girth (CG), 36.88 cm for cannon length (CL), 83.53 cm for leg length (LL), and 8.31 cm for hoof height (HH). Head measurements averaged 54.76 cm for head length (HL) and 21.53 cm for head width (HdW). Among all traits, body length exhibited the greatest absolute variability (SD = 8.21), followed by heart girth (SD = 8.07) and withers height (SS = 7.15) The coefficients of variation ranged from 4.17 for tailhead height (TH) to 18.33 for hoof height (HH). The observed ranges indicated substantial phenotypic variation across the evaluated morphometric traits (Table 2 and Table 3).

Afghan-origin horses tended to exhibit greater values for several body measurements than the indigenous Bursa and Nazilli populations, suggesting a larger body frame. With the exception of ischial tuberosity height, the significant differences observed among breeding origins were largely associated with the comparatively larger body size of the Afghan-origin horses, whereas the Bursa and Nazilli populations exhibited broadly similar morphometric characteristics. In addition, body length was positively correlated with several morphometric traits, including heart girth. These relationships should be interpreted as descriptive morphometric associations within the studied population (Table 4).

Breeding origin significantly affected several body measurements, including WH, BL, and IW (*p* < 0.05), as well as TH (*p* < 0.001) and HW, CD, CW, and HG (*p* < 0.01). In contrast, CH, BH, and ITH did not differ significantly among breeding-origin groups (*p* > 0.05). Among the limb traits, significant effects of breeding origin were detected for CG (*p* < 0.05) and CL (*p* < 0.001), whereas HH and LL were not significantly influenced (*p* > 0.05). Afghan- and Nazilli-origin horses showed comparable values for cannon girth and cannon length, whereas Bursa horses exhibited significantly lower values for both measurements. For head measurements, significant differences were observed for HL (*p* < 0.05) and HdW (*p* < 0.001). Afghan and Nazilli horses displayed similar head dimensions, whereas Bursa horses had significantly smaller head measurements than the other two groups (Table 4).

Age-specific analyses within each breeding-origin group were not performed due to limited sample sizes in some subclasses. Nevertheless, the overall distribution of horses among age groups enabled the evaluation of age-related changes in morphometric characteristics (Table 5).

The significant age-related differences were primarily attributable to horses in the 0–3-year age group, representing the active growth phase. Highly significant age effects were detected for WH, BL, CD, CW, and HG (*p* < 0.001), whereas TH was affected at *p* < 0.01. Significant age-related differences were also observed for CH, BH, ITH, IW, and HW (*p* < 0.05). In contrast, no significant differences in WH, CH, BH, BL, CD, CW, HG, or HW were detected among horses aged 4 years and older (*p* > 0.05).

Age also significantly influenced limb measurements. CG showed the strongest age effect (*p* < 0.001), although CL (*p* < 0.05), HH (*p* < 0.01), and LL (*p* < 0.05) were also affected by age. Horses in the 4–6, 7–9, and ≥10-year age groups exhibited similar values for cannon girth, cannon length, and hoof height, whereas horses aged 0–3 years differed significantly from the older groups, indicating age-related differences in these limb measurements within the studied population. Head measurements likewise exhibited age-related variation. Horses aged four years and older showed similar HL, whereas the 0–3-year age group had significantly smaller values (*p* < 0.05). A comparable pattern was observed for HdW, although Tukey’s multiple comparison test indicated no significant differences between the 0–3- and 7–9-year groups or between the 4–6- and ≥10-year groups.

Sex significantly affected several morphometric traits, although most body measurements did not differ between males and females. Significant differences were observed for TH, CD, CW (*p* < 0.01), and HG (*p* < 0.05), with males exhibiting larger values than females. In contrast, WH, CH, BH, BL, IW, and HW were not significantly influenced by sex (Table 6).

Sex was associated with variation in all evaluated limb measurements. Males generally exhibited greater values for CG, CL, HH, and LL than females, with the strongest effect observed for CL (*p* < 0.001), followed by CG and HH (*p* < 0.01), and LL (*p* < 0.05). Head measurements were also significantly affected by sex. Male horses exhibited greater HL and HdW than females, with significant differences for head length (*p* < 0.01) and highly significant differences for head width (*p* < 0.001) (Table 6).

The Pearson correlation coefficients among the evaluated morphometric traits are presented in Table 7. The strongest correlation was observed between CH and BH (*r* = 0.920, *p* < 0.001), followed by WH and BH (*r* = 0.873, *p* < 0.001) and WH and CH (*r* = 0.860, *p* < 0.001). ITH was also positively correlated with BH (*r* = 0.825, *p* < 0.001), CH (*r* = 0.808, *p* < 0.001), and WH (*r* = 0.744, *p* < 0.001). Among body measurements, CD was positively correlated with TH (*r* = 0.673, *p* < 0.001) and BL (*r* = 0.610, *p* < 0.001). HG was also positively correlated with CD (*r* = 0.609, *p* < 0.001). BL showed positive correlations with TH (*r* = 0.577, *p* < 0.001), CW (*r* = 0.572, *p* < 0.001), CG (*r* = 0.570, *p* < 0.001), and HL (*r* = 0.575, *p* < 0.001). Most of the remaining correlations were weak to moderate. No significant correlations were observed between HdW and WH, TH, IW, or LL, or between CL and HW or CG. Similarly, HH was not significantly correlated with CL or HdW, with the correlation between HH and CL being slightly negative (*r* = −0.041, *p* > 0.05). (Table 7).

## 4. Discussion

The morphometric values observed in the present study were generally within or close to the ranges previously reported for indigenous Turkish horse populations [9,10,15,16,19,22,23], although back height and body length were slightly greater than some previously reported values. Horses raised under different ecological and breeding conditions may exhibit variation in morphometric characteristics [24]. However, because management and environmental effects were not independently evaluated in the present study, their potential contribution to the observed differences cannot be determined. Ribeiro et al. [13] reported significant effects of sex on several morphometric traits, including withers height, croup height, chest depth, body length, and cannon circumference. The present findings are generally consistent with these observations, as males tended to exhibit greater values for several morphometric measurements than females, particularly for some body and limb traits. Thus, sex-related variation may be considered when describing and comparing the morphometric characteristics of horse populations. Overall, the similarities with previously reported values should be regarded as descriptive morphometric observations rather than as evidence of a characteristic or standardized conformation, because formal comparisons with breed standards and body proportionality indices were not performed.

Among the evaluated morphometric measurements, withers height (WH) is commonly used to describe overall body size in horses. Rogers et al. [14] reported that Thoroughbred horses attain approximately 98% of their mature withers height by 24 months of age, while Batu [9] indicated that changes in skeletal height and body dimensions may continue until approximately four to five years of age. In the present cross-sectional study, no significant differences in WH were observed among horses older than four years. Because individual horses were measured only once, this finding should not be interpreted as evidence that growth was completed at this age, but rather as indicating limited differences in WH among the older age groups included in the study. Previous studies have indicated that morphometric traits such as WH may be influenced by both genetic and environmental factors, including nutrition and management conditions [9]. However, these factors were not independently evaluated in the present study, and their contribution to the observed morphometric variation cannot be determined. Therefore, the present findings provide primarily descriptive information on age-related variation in WH within the studied population.

The high positive correlations observed among CH, BH, and WH (*r* = 0.860–0.920, all *p* < 0.001) indicate close associations among these height measurements in the horses examined. ITH was also positively associated with BH (*r* = 0.825), CH (*r* = 0.808), and WH (*r* = 0.744) (all *p* < 0.001). These relationships provide descriptive baseline information on associations among body height measurements in the studied population. Previous studies have discussed croup conformation in relation to locomotor characteristics [9]. However, locomotor function and body proportionality indices were not evaluated in the present study. Therefore, the relationships among these height measurements should be interpreted primarily as morphometric associations within the studied population.

BL, CD, CW, and HG are commonly used morphometric traits for describing body size. Afghan-origin horses generally exhibited higher values for several body measurements than the indigenous Turkish populations, reflecting morphometric differences within the studied sample. Body length was positively correlated with several morphometric traits, particularly chest depth, tail height, head length, chest width, and cannon girth. A positive correlation was also observed between heart girth and chest depth (*r* = 0.609, *p* < 0.001), indicating an association between these two morphometric measurements within the studied population. These findings are generally consistent with those of Padilha et al. [25], who reported positive associations among croup height, body length, head dimensions, and other skeletal measurements. However, the Pearson correlation coefficients in the present study were calculated across the overall study population without adjustment for breeding origin, age, or sex. Accordingly, these correlations should be interpreted primarily as descriptive morphometric associations.

Limb and head measurements also contributed to the morphometric characterization of the studied horses. These measurements showed varying patterns in relation to breeding origin, age, and sex, with the magnitude and statistical significance of the differences differing among traits. Cannon girth (CG) has been discussed in the literature in relation to skeletal robustness [9,10]. In the present study, CG showed positive correlations with several morphometric traits, indicating associations between cannon girth and other body measurements within the studied population. Ribeiro et al. [13] similarly reported greater cannon circumference in males than in females and discussed this finding in relation to sexual dimorphism in skeletal characteristics. In the present study, the observed differences and associations involving cannon measurements provide additional descriptive information on limb morphology. Sex-related differences were significant for CG and HH (*p* < 0.01), CL (*p* < 0.001), and LL (*p* < 0.05), indicating that the statistical patterns differed among individual limb traits.

Regarding head morphology, head length in the present study was greater than the values reported in some previous studies on Turkish horse populations [16,26,27]. However, because formal comparisons with breed standards and body proportionality indices were not performed, this difference should not be interpreted as evidence of a characteristic feature of Turkish ambling horses. Further comparative studies involving appropriate reference populations and statistical comparisons would be useful for determining whether the observed differences represent consistent morphometric characteristics of this population. Thus, the head measurements obtained in the present study may be considered complementary information for the phenotypic characterization of the studied horses.

This study has several limitations that should be considered when interpreting the findings. Although the overall sample included 102 horses, the distribution of animals among breeding-origin and age groups was unbalanced, particularly because the Afghan-origin group included only nine horses and some age groups were represented by relatively few animals. In addition, the horses were not selected through random sampling. Breeding origin, geographic region, farm, and management conditions could not be fully separated within the observational design of the study. Therefore, the observed differences among breeding-origin groups should be interpreted with caution and should not necessarily be attributed solely to breeding origin.

Residual normality, homogeneity of variance, and potential outliers were examined as part of the assessment of model assumptions. Residual normality was acceptable for 7 of the 17 evaluated traits, while homogeneity of variance was observed for 7 traits; both assumptions were satisfied simultaneously for TH, BL, and CW. The number of potential outliers was low, ranging from 0 to 2 observations per trait. Departures from residual normality and/or homogeneity of variance were observed for some morphometric traits. Given the unbalanced sample structure and the relatively small number of observations in some subgroups, these departures should be considered when interpreting the corresponding model results. Accordingly, findings for traits that did not fully meet the model assumptions were interpreted with appropriate caution.

Pearson correlation coefficients were calculated across the overall study population without adjustment for breeding origin, age, or sex and should therefore be regarded primarily as descriptive measures of association among the evaluated morphometric traits. Furthermore, the ambling gait was used as an inclusion criterion and was not quantitatively evaluated in terms of gait performance or biomechanics. Future studies involving larger and more balanced populations, together with longitudinal morphometric observations, quantitative gait assessments, and molecular genetic analyses, would be useful for further evaluating the phenotypic variation observed in Turkish ambling horses.

## 5. Conclusions

This study characterized the morphometric traits of Turkish ambling horses according to breeding origin, age, and sex and provides baseline phenotypic information on the studied population. Morphometric differences were observed among breeding-origin, age, and sex groups, and positive associations were identified among several major body measurements. These findings contribute to the phenotypic characterization of Turkish ambling horses and may provide baseline information for future studies. However, the unbalanced distribution of animals among groups, particularly the small number of Afghan-origin horses, as well as the observational and non-random sampling design, should be considered when interpreting the observed differences. Further studies involving larger and more balanced populations, together with quantitative gait assessment and molecular genetic analyses, would be valuable for further characterizing the phenotypic and genetic variation of Turkish ambling horses.

## Figures and Tables

**Table 1:** Combined distribution of horses according to breeding origin, age group, and sex.

Breeding Origin	Age Group (Years)	Female (*n*)	Male (*n*)	Total (*n*)
Afghan-origin	0–3	0	2	2
	4–6	0	2	2
	7–9	0	1	1
	≥10	0	4	4
	Total	0	9	9
Bursa indigenous	0–3	4	6	10
	4–6	3	2	5
	7–9	4	1	5
	≥10	6	2	8
	Total	17	11	28
Nazilli indigenous	0–3	7	11	18
	4–6	3	21	24
	7–9	2	6	8
	≥10	8	7	15
	Total	20	45	65
Overall total	—	37	65	102

**Table 2:** Descriptive statistics of the morphometric body measurements (cm) of Turkish ambling horses (*n* = 102).

Trait	$\bar{X}$	$S_{\bar{x}}$	SD	CK	Range	Min	Max
WH	143.72	0.71	7.15	5.16	52	117	169
CH	134.44	0.65	6.63	4.93	48.5	116	164.5
BH	135.76	0.63	6.43	4.73	51	116	167
TH	128.68	0.53	5.37	4.17	28.5	112	140.5
ITH	123.11	0.67	6.81	5.53	58	103	161
BL	137.56	0.81	8.21	5.96	42	116	158
CD	64.53	0.41	4.10	6.35	21	55	76
CW	34.84	0.41	4.22	12.13	23	25	48
HG	156.92	0.79	8.07	5.14	44	136	180
IW	22.68	0.19	2.01	8.83	11	18	29
HW	41.66	0.43	4.40	10.57	23	29	52
CG	18.81	0.21	2.11	11.24	16	8	24
CL	36.88	0.29	3.01	8.14	13	29	42
HL	54.76	0.29	2.98	5.44	14	48	62
HdW	21.53	0.17	1.78	8.30	10	15	25
HH	8.31	0.15	1.52	18.33	8	4	12
LL	83.53	0.49	4.96	5.94	28	72	100

$\bar{X}$, mean; $S_{\bar{x}}$, standard error of the mean; SD, standard deviation; CK, coefficient of variation; Min, minimum; Max, maximum. Withers height (WH), croup height (CH), back height (BH), tailhead height (TH), ischial tuberosity height (ITH), body length (BL), chest depth (CD), chest width (CW), heart girth (HG), ischial width (IW), hip width (HW), cannon girth (CG), cannon length (CL), head length (HL), head width (HdW), hoof height (HH), and leg length (LL).

**Table 3:** Assessment of model assumptions and potential outliers for the evaluated morphometric traits.

Trait	Shapiro–Wilk *p*	Residual Normality	Levene *p*	Variance Homogeneity	Potential Outliers (*n*)
**WH**	<0.001	Departure	<0.001	Heterogeneous	2
**CH**	<0.001	Departure	0.003	Heterogeneous	2
**BH**	<0.001	Departure	0.005	Heterogeneous	2
**TH**	0.848	Acceptable	0.270	Homogeneous	0
**ITH**	<0.001	Departure	0.010	Heterogeneous	2
**BL**	0.803	Acceptable	0.065	Homogeneous	0
**CD**	0.163	Acceptable	0.027	Heterogeneous	1
**CW**	0.187	Acceptable	0.123	Homogeneous	0
**HG**	0.473	Acceptable	0.008	Heterogeneous	1
**IW**	0.665	Acceptable	0.009	Heterogeneous	1
**HW**	<0.001	Departure	0.414	Homogeneous	2
**LL**	0.015	Departure	0.286	Homogeneous	1
**CG**	<0.001	Departure	0.002	Heterogeneous	2
**CL**	0.004	Departure	0.147	Homogeneous	1
**HL**	0.283	Acceptable	0.002	Heterogeneous	0
**HdW**	0.004	Departure	0.072	Homogeneous	2
**HH**	0.493	Acceptable	0.023	Heterogeneous	1

Assessment of model assumptions indicated that residual normality was acceptable for 7 of the 17 morphometric traits, while homogeneity of variance was observed for 7 traits. Both assumptions were satisfied simultaneously for TH, BL, and CW. The number of potential outliers ranged from 0 to 2 observations per trait. Detailed results of the model assumption assessments are presented in Table 3.

**Table 4:** Least squares means ± standard error of morphometric traits according to breeding origin in Turkish ambling horses.

Trait	Afghan (*n* = 9)	Bursa (*n* = 28)	Nazilli (*n* = 65)	*p*-Value
Body measurements				
WH	148.5 ± 2.25 a	144.12 ± 2.03 ab	142.89 ± 0.58 b	*
CH	135.38 ± 1.42	133.48 ± 2.01	134.73 ± 0.54	
BH	136.44 ± 1.41	134.26 ± 1.91	136.31 ± 0.52	
TH	129.27 ± 1.46 a	125.23 ± 1.11 b	130.08 ± 0.57 a	***
ITH	122.50 ± 1.30	122.07 ± 1.91	123.65 ± 0.64	
BL	144.11 ± 2.26 a	136.21 ± 1.65 b	137.24 ± 0.97 b	*
CD	68.67 ± 0.95 a	62.69 ± 0.91 c	64.75 ± 0.42 b	**
CW	38.67 ± 1.30 a	33.30 ± 0.78 b	34.98 ± 0.49 b	**
HG	163.22 ± 2.80 a	154.30 ± 1.73 b	157.17 ± 0.87 b	**
IW	23.89 ± 0.53 a	23.25 ± 0.47 a	22.27 ± 0.21 b	*
HW	46.22 ± 0.99 a	41.32 ± 1.06 b	41.18 ± 0.44 b	**
Limb measurements				
CG	19.50 ± 0.73 a	17.89 ± 0.51 b	19.10 ± 0.21 a	*
CL	38.11 ± 0.58 a	35.00 ± 0.64 b	37.52 ± 0.32 a	***
HH	8.22 ± 0.27	7.82 ± 0.32	8.53 ± 0.18	
LL	85.66 ± 1.26	82.50 ± 1.02	83.69 ± 0.60	
Head measurements				
HL	56.66 ± 1.05 a	53.50 ± 0.55 b	55.04 ± 0.34 a	*
HdW	22.88 ± 0.38 a	20.39 ± 0.44 b	21.84 ± 0.15 a	***

Different letters indicate significant differences among groups (*p* < 0.05), * *p* < 0.05; **, *p* < 0.01; ***, *p* < 0.001.

**Table 5:** Least squares means ± standard error of morphometric traits according to age in Turkish ambling horses.

Trait	0–3 yr (*n* = 30)	4–6 yr (*n* = 31)	7–9 yr (*n* = 14)	≥10 yr (*n* = 27)	*p*-Value
Body measurements					
WH	140.66 ± 0.90 b	145.51 ± 0.78 a	146.60 ± 2.13 a	145.50 ± 3.75 a	***
CH	132.15 ± 0.76 b	136.01 ± 0.88 a	136.32 ± 2.24 a	139.12 ± 3.89 a	*
BH	133.60 ± 0.74 b	137.14 ± 0.89 a	137.42 ± 2.01 a	140.75 ± 3.90 a	*
TH	126.97 ± 0.71 b	130.98 ± 0.96 a	128.82 ± 1.38 ab	129.93 ± 1.76 ab	**
ITH	121.03 ± 0.74 b	124.30 ± 0.98 ab	124.53 ± 2.01 ab	128.81 ± 4.64 a	*
BL	133.06 ± 1.02 b	140.87 ± 1.14 a	141.50 ± 1.96 a	145.50 ± 2.45 a	***
CD	62.30 ± 0.47 b	66.61 ± 0.67 a	65.96 ± 1.11 a	67.62 ± 0.86 a	***
CW	32.72 ± 0.45 b	36.12 ± 0.65 a	37.14 ± 1.23 a	38.87 ± 1.74 a	***
HG	152.36 ± 1.08 b	161.20 ± 2.07 a	160.07 ± 1.68 a	162.68 ± 2.10 a	***
IW	22.10 ± 0.25 b	23.16 ± 0.37 a	22.92 ± 0.48 ab	24.00 ± 0.84 a	*
HW	40.10 ± 0.61 b	42.90 ± 0.60 a	43.35 ± 1.51 a	43.50 ± 1.16 a	*
Limb measurements					
CG	17.61 ± 0.27 b	19.54 ± 0.24 a	20.39 ± 0.44 a	20.50 ± 0.76 a	***
CL	36.08 ± 0.41 b	38.22 ± 0.46 a	36.14 ± 0.84 ab	37.87 ± 1.17 ab	*
HH	7.69 ± 0.21 b	9.04 ± 0.21 a	8.35 ± 0.41 ab	9.12 ± 0.54 a	**
LL	82.22 ± 0.69 b	84.67 ± 0.83 ab	82.71 ± 1.18 b	88.62 ± 1.32 a	*
Head measurements					
HL	53.71 ± 0.34 b	55.54 ± 0.53 a	55.78 ± 0.82 a	56.37 ± 1.42 a	*
HdW	21.00 ± 0.22 b	22.32 ± 0.23 a	21.00 ± 0.67 b	22.75 ± 0.59 a	*

Different letters indicate significant differences among groups (*p* < 0.05) *, *p* < 0.05; **, *p* < 0.01; ***, *p* < 0.001.

**Table 6:** Least squares means ± standard error of morphometric traits according to sex in Turkish ambling horses.

Trait	Female *(n* = 37)	Male (*n* = 65)	*p*-Value
Body measurements			
WH	142.90 ± 1.56	144.21 ± 0.64	
CH	133.90 ± 1.41	134.76 ± 0.63	
BH	135.15 ± 1.44	136.12 ± 0.55	
TH	126.59 ± 0.92	129.92 ± 0.60	**
ITH	121.82 ± 1.54	123.88 ± 0.55	
BL	135.86 ± 1.55	138.57 ± 0.89	
CD	62.98 ± 0.66	65.45 ± 0.48	**
CW	32.75 ± 0.64	36.09 ± 0.48	**
HG	154.52 ± 1.55	158.34 ± 0.83	*
IW	22.84 ± 0.39	22.59 ± 0.21	
HW	40.89 ± 0.81	42.12 ± 0.49	
Limb measurements			
CG	17.94 ± 0.37	19.32 ± 0.22	**
CL	35.57 ± 0.47	37.65 ± 0.34	***
HH	7.76 ± 0.25	8.63 ± 0.17	**
LL	82.00 ± 0.86	84.45 ± 0.56	*
Head measurements			
HL	53.55 ± 0.45	55.48 ± 0.35	**
HdW	20.55 ± 0.33	22.12 ± 0.16	***

*, *p* < 0.05; **, *p* < 0.01; ***, *p* < 0.001.

**Table 7:** Phenotypic correlation coefficients among morphometric traits in Turkish ambling horses.

Trait	WH	CH	BH	TH	ITH	BL	CD	CW	HG	IW	HW	LL	CG	CL	HL	HdW	HH
WH	1.000																
CH	0.860 (<0.0001)	1.000															
BH	0.873 (<0.0001)	0.920 (<0.0001)	1.000														
TH	0.509 (<0.0001)	0.526 (<0.0001)	0.473 (<0.0001)	1.000													
ITH	0.744 (<0.0001)	0.808 (<0.0001)	0.825 (<0.0001)	0.464 (<0.0001)	1.000												
BL	0.479 (<0.0001)	0.488 (<0.0001)	0.456 (<0.0001)	0.577 (<0.0001)	0.454 (<0.0001)	1.000											
CD	0.454 (<0.0001)	0.438 (<0.0001)	0.414 (<0.0001)	0.673 (<0.0001)	0.342 (0.000)	0.610 (<0.0001)	1.000										
CW	0.418 (<0.0001)	0.450 (<0.0001)	0.388 (<0.0001)	0.512 (<0.0001)	0.408 (<0.0001)	0.572 (<0.0001)	0.486 (<0.0001)	1.000									
HG	0.448 (<0.0001)	0.393 (<0.0001)	0.407 (<0.0001)	0.564 (<0.0001)	0.350 (0.000)	0.535 (<0.0001)	0.609 (<0.0001)	0.310 (0.002)	1.000								
IW	0.367 (0.000)	0.354 (0.000)	0.327 (0.001)	0.337 (0.001)	0.307 (0.002)	0.373 (0.000)	0.465 (<0.0001)	0.219 (0.027)	0.456 (<0.0001)	1.000							
HW	0.345 (0.000)	0.302 (0.002)	0.351 (0.000)	0.354 (0.000)	0.291 (0.003)	0.376 (<0.0001)	0.459 (<0.0001)	0.256 (0.010)	0.380 (<0.0001)	0.482 (<0.0001)	1.000						
LL	0.531 (<0.0001)	0.493 (<0.0001)	0.530 (<0.0001)	0.446 (<0.0001)	0.475 (<0.0001)	0.450 (<0.0001)	0.389 (<0.0001)	0.374 (0.000)	0.329 (0.001)	0.153 (0.125)	0.275 (0.005)	1.000					
CG	0.467 (<0.0001)	0.425 (<0.0001)	0.393 (<0.0001)	0.383 (<0.0001)	0.335 (0.001)	0.570 (<0.0001)	0.452 (<0.0001)	0.317 (0.001)	0.339 (0.001)	0.297 (0.002)	0.309 (0.002)	0.288 (0.003)	1.000				
CL	0.225 (0.023)	0.260 (0.008)	0.214 (0.031)	0.392 (<0.0001)	0.213 (0.032)	0.389 (<0.0001)	0.305 (0.002)	0.504 (<0.0001)	0.200 (0.044)	0.213 (0.031)	0.069 (0.489)	0.266 (0.007)	0.063 (0.530)	1.000			
HL	0.319 (0.001)	0.283 (0.004)	0.256 (0.009)	0.513 (<0.0001)	0.220 (0.026)	0.575 (<0.0001)	0.571 (<0.0001)	0.396 (<0.0001)	0.442 (<0.0001)	0.293 (0.003)	0.325 (0.001)	0.375 (0.000)	0.262 (0.008)	0.351 (0.000)	1.000		
HdW	0.175 (0.079)	0.234 (0.018)	0.273 (0.006)	0.184 (0.064)	0.230 (0.020)	0.448 (<0.0001)	0.300 (0.002)	0.418 (<0.0001)	0.258 (0.009)	0.191 (0.054)	0.235 (0.017)	0.186 (0.061)	0.256 (0.009)	0.398 (<0.0001)	0.293 (0.003)	1.000	
HH	0.557 (<0.0001)	0.507 (<0.0001)	0.499 (<0.0001)	0.291 (0.003)	0.488 (<0.0001)	0.445 (<0.0001)	0.308 (0.002)	0.320 (0.001)	0.346 (0.000)	0.415 (<0.0001)	0.262 (0.008)	0.429 (<0.0001)	0.432 (<0.0001)	−0.041 (0.680)	0.341 (0.001)	0.061 (0.545)	1.000

## Data Availability

All data generated or analyzed during this study are included in this published article. The datasets generated and/or analyzed during the current study are available from the corresponding author upon reasonable request.
